# Impact of statin therapy on mortality in patients with sepsis-associated acute respiratory distress syndrome (ARDS) depends on ARDS severity: a prospective observational cohort study

**DOI:** 10.1186/s12916-015-0368-6

**Published:** 2015-06-01

**Authors:** Ashham Mansur, Maximilian Steinau, Aron Frederik Popov, Michael Ghadimi, Tim Beissbarth, Martin Bauer, José Hinz

**Affiliations:** Department of Anesthesiology, University Medical Center, Georg August University, D-37075 Goettingen, Germany; Department of Cardiothoracic Transplantation & Mechanical Support, Royal Brompton and Harefield Hospital, Harefield, Hill End Road, UB9 6JH London, UK; Department of General and Visceral Surgery, University Medical Center, Georg August University, D-37075 Goettingen, Germany; Department of Medical Statistics, University Medical Center, Georg August University, D-37075 Goettingen, Germany

**Keywords:** 3-hydroxy-3-methylglutaryl CoA reductase inhibitor, 28-day survival, Acute respiratory distress syndrome, Intensive care unit, Statins

## Abstract

**Background:**

Previous investigations have presumed a potential therapeutic effect of statin therapy in patients with acute respiratory distress syndrome (ARDS). Statins are expected to attenuate inflammation in the lungs of patients with ARDS due to their anti-inflammatory effects. Clinical investigations of the role of statin therapy have revealed contradictory results. This study aimed to investigate whether pretreatment and continuous therapy with statins in patients with sepsis-associated ARDS are associated with 28-day survival according to disease severity (mild, moderate, or severe).

**Methods:**

Patients with sepsis-associated ARDS from the surgical intensive care were enrolled in this prospective observational investigation. ARDS was classified into three groups (mild, moderate, and severe); 28-day mortality was recorded as the primary outcome variable and organ failure was recorded as secondary outcome variable. Sequential Organ Failure Assessment scores and the requirements for organ support were evaluated throughout the observational period to assess organ failure.

**Results:**

404 patients with sepsis-associated ARDS were enrolled in this investigation. The distribution of the ARDS subgroups was 13 %, 59 %, and 28 % for mild, moderate, and severe disease, respectively. Statin therapy improved 28-day survival exclusively in the patients with severe ARDS compared with patients without statin therapy (88.5 % and 62.5 %, respectively; *P* = 0.0193). To exclude the effects of several confounders, we performed multivariate Cox regression analysis, which showed that statin therapy remained a significant covariate for mortality (hazard ratio, 5.46; 95 % CI, 1.38–21.70; *P* = 0.0156). Moreover, after carrying a propensity score-matching in the severe ARDS cohort, Kaplan-Meier survival analysis confirmed the improved 28-day survival among patients with statin therapy (*P* = 0.0205). Patients with severe ARDS who received statin therapy had significantly more vasopressor-free days compared with those without statin therapy (13 ± 7 and 9 ± 7, respectively; *P* = 0.0034), and they also required less extracorporeal membrane oxygenation (ECMO) therapy and had more ECMO-free days (18 ± 9 and 15 ± 9, respectively; *P* = 0.0873).

**Conclusions:**

This investigation suggests a beneficial effect of continuous statin therapy in patients with severe sepsis-associated ARDS and a history of prior statin therapy. Further study is warranted to elucidate this potential effect.

**Electronic supplementary material:**

The online version of this article (doi:10.1186/s12916-015-0368-6) contains supplementary material, which is available to authorized users.

## Background

Despite improvements in supportive treatment for acute respiratory distress syndrome (ARDS), mortality among patients with sepsis-associated ARDS remains high [1]. The pathogenesis of this syndrome is characterized by overwhelming inflammation that results in alveolar damage, accompanied by the exudation of protein-rich pulmonary-edema fluid in the alveolar space, which leads to respiratory failure [2]. During the course of this inflammation, which results in cellular damage and death, the development of both pulmonary and extra-pulmonary organ failure is promoted. Consequently, therapies that attenuate inflammation may have positive impacts on the clinical course and outcome of patients with ARDS [3]. Inhibitors of 3-hydroxy-3-methylglutaryl coenzyme A reductase, known as statins, are frequently prescribed for treatment of hypercholesterolemia worldwide. However, statins also have immunosuppressive properties and can attenuate inflammation. In ARDS murine models, statins have been shown to prevent disease development [4, 5]. Simvastatin, which is among the most prescribed statins worldwide, has been shown to reduce pulmonary and systemic inflammatory responses in a human model of ARDS induced by lipopolysaccharide inhalation [6]. Whereas several studies have suggested that patients with severe inflammatory conditions, such as sepsis or ARDS, who receive statins have improved clinical outcomes [4, 6–10], other studies examining the impacts of statin therapy on the clinical outcomes of these patients have failed to show any beneficial effects [11–13]. These contradictory results regarding the beneficial impacts of statin therapy on ARDS patients might be due to the fact that the previous studies evaluated heterogenic ARDS patient groups without considering disease severity as a potential determinant of therapeutic responsiveness.

According to the Berlin definition of ARDS [14], disease severity can be classified based on the degree of arterial hypoxemia, as indicated by the P_a_O_2_/F_i_O_2_ ratio (P/F), as follows: mild, P/F of 201 to 300 mmHg; moderate, P/F of 101 to 200 mmHg; and severe, P/F of <100 mmHg.

Based on this knowledge, we conducted a prospective observational study to test the hypothesis that statin therapy improves the clinical course of sepsis-associated ARDS depending on disease severity (mild, moderate, or severe).

## Methods

### Patients

This study was approved by the University of Goettingen ethics committee in Goettingen, Germany (1/15/12) and conformed to the ethical principles of the Declaration of Helsinki. Written informed consent was obtained from all patients or their legal representatives. Adult Caucasian patients admitted to the surgical intensive care units (ICUs) of the University Medical Center of Goettingen between April 2012 and September 2014 were evaluated daily according to the American College of Chest Physicians/Society of Critical Care Medicine (ACCP/SCCM) criteria for sepsis [15, 16]. Patients with sepsis were screened daily according to the Berlin definition of ARDS to identify those with sepsis-associated ARDS [14–16]. The patient exclusion criteria were as follows: those who i) were younger than 18 years of age; ii) were pregnant or nursing an infant; iii) were receiving immunosuppressive therapy; iv) had a documented myocardial infarction within the previous 6 weeks; v) had New York Heart Association functional class IV chronic heart failure; vi) were infected with human immunodeficiency virus; vii) had a do not resuscitate or do not treat order; viii) were not expected to survive the next 28 days because of an uncorrectable medical condition (e.g., poorly controlled neoplasm); ix) were in a chronic vegetative state with pronounced neurological impairment; x) were currently participating in any clinical trial (of a drug or device); xi) could not be fully evaluated during the study period; and xii) were a study-site employee or the family member of a study-site employee. Because interracial genetic differences may affect the clinical courses of infectious diseases, we exclusively recruited Caucasians for this observational study. Caucasians also comprise the greatest proportion of patients admitted to our surgical ICUs. According to pharmacogenetic studies, the frequency of polymorphisms in statin transporter genes vary markedly between populations and can have profound effects on statin pharmacokinetics [17, 18]. In particular, a common genetic variant of organic anion-transporting polypeptide 1B1 with a diverse distribution among populations reduces the hepatic uptake of many statins, increasing the risks of statin-induced myopathy and adverse events [18, 19].

In our tertiary medical center (a member of the German ARDS network), the treatment goals for ARDS patients consist of supportive care and a protective strategy of lung ventilation using low tidal volumes to limit end-inspiratory plateau pressure. A tidal volume of 6 cc/kg predicted body weight or lower is used to maintain an inspiratory plateau pressure of <30 cm of water [20]. Patients with more severe hypoxemia exhibit a high positive end-expiratory pressure [21]. Patients with refractory hypoxemia are placed in the prone position to improve oxygenation [22, 23]. Additionally, rescue oxygenation is performed at our center using standardized extracorporeal membrane oxygenation (ECMO) therapy for patients with profound refractory hypoxemia [24]. ARDS patients also receive conservative intravenous fluid management to reduce pulmonary microvascular pressure and the likelihood of developing pulmonary edema [25].

### Data collection

Death within 28 days of sepsis onset was recorded as the primary outcome variable. Two morbidity scores, the Sequential Organ Failure Assessment (SOFA) [26] and Acute Physiology and Chronic Health Evaluation (APACHE) II [27] scores, were evaluated at sepsis onset. Organ function was reassessed using SOFA scores over 28 days in the ICU to monitor morbidity. Organ support-free days (mechanical ventilation, vasopressor therapy, renal replacement therapy, and ECMO therapy) and the length of ICU stay were recorded as secondary outcome variables. The baseline that was used for support-free days was the length of ICU stay for each patient. Clinical data were collected from the electronic patient record system (IntelliSpace Critical Care and Anesthesia (ICCA); Philips Healthcare, Andover, Massachusetts, USA). All medical records, including microbiological findings and medication histories, were obtained from these electronic health records. Prior statin therapy and comorbidities were identified by examining physicians’ notes, through anamnestic questionnaires of the patients or their legal representatives, and by consulting each patient’s family doctor.

### Statistical analyses

Statistical analyses were performed using Statistica software (version 10; StatSoft, Tulsa, Oklahoma, USA). The significance of the categorical variables was calculated using two-sided Fisher’s exact or χ^2^ tests, as appropriate. Two continuous variables were compared using the Mann-Whitney test. Time-to-event data were compared using the log-rank test with Statistica package for Kaplan-Meier survival analysis. A power calculation was conducted using Statistica package for power analysis. To exclude the effects of potential confounders (age, gender and body mass index (BMI)) and covariates that varied at baseline (e.g., comorbidities and recent surgical history) on survival, we performed multivariate Cox regression analysis to examine survival time. A *P* value of <0.05 was considered statistically significant. Propensity score matching was performed using the statistical computing software R (version 3.1.1) with MatchIt package (version 2.4-21).

## Results

### Patients and baseline characteristics

**Fig. 1:**
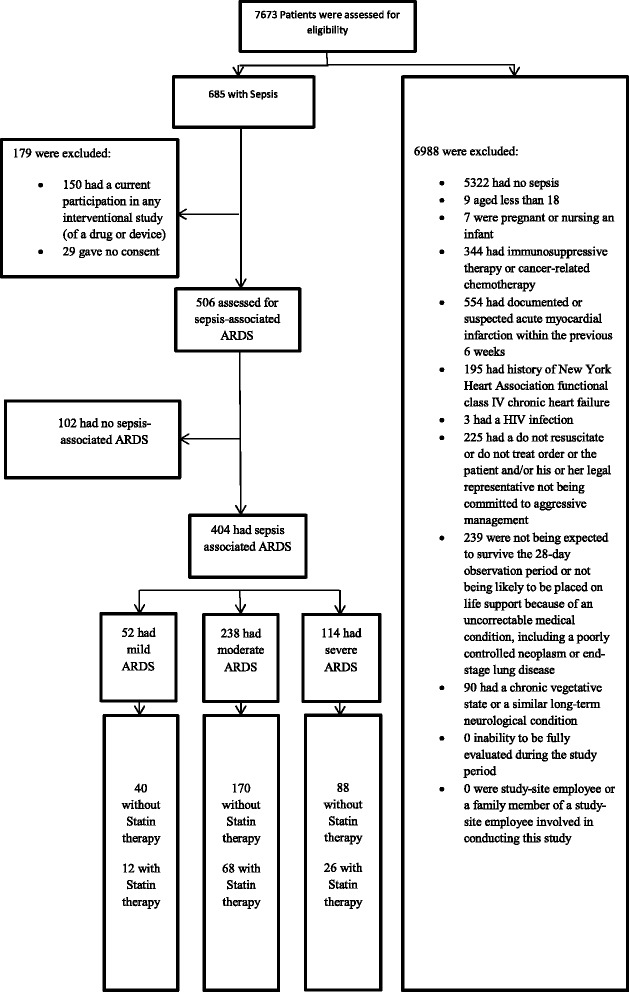
Population of patients who were screened and followed-up

A total of 404 patients with sepsis developed ARDS and were enrolled in this study (Fig. 1). The distribution of the ARDS subgroups was 13 %, 59 %, and 28 % for mild, moderate, and severe ARDS, respectively. Among all of the patients, 27 % were pretreated with statins, and statin therapy was continued over the observation period in this patient group. Most of the patients in the statin group were pretreated with simvastatin (87.1 %, Table 1), which was given at the same dose after admission. The patients who were pretreated with one of the other statins were switched to simvastatin (the standard statin in our ICUs) at an equivalent dose. Patients who were fed via a tube were still given statins. The patients underwent statin therapy because of associated comorbid conditions. The rate of statin therapy did not differ significantly among the three ARDS subgroups. Simvastatin (20 or 40 mg) was the most frequently used statin (87.1 %; Table 1). The ages of the patients ranged from 19 to 92 years (median, 63 years; Table 1). The ARDS patients on statin therapy were significantly older than those who were not on this therapy (70 ± 11 and 60 ± 16, respectively; *P* <0.001; Table 1). No differences were recorded in gender or BMI between the two groups (Table 1). The proportion of patients with septic shock was significantly higher among the patients without statin therapy compared with those receiving this therapy (68 % and 52 %, respectively; *P* = 0.0035). At baseline, the patients without statin therapy had significantly higher SOFA scores compared with those receiving therapy (9.9 ± 3.8 and 8.9 ± 3.4, respectively; *P* = 0.0158). No differences were found in APACHE II scores with respect to statin therapy at baseline (Table 1).

**Table 1:** Patients’ baseline characteristics according to statin usage

	All	ARDS		*P* value
	(n = 404)	without statins	with statins	
		(n = 296)	(n = 108)	
Age, years	63 ± 15	60 ± 16	70 ± 11	<0.0001
Male, %	68	65	76	0.0533
Body mass index	28 ± 7	28 ± 8	28 ± 5	0.4304
Severity of sepsis				
Sepsis/severe sepsis, %	36	32	48	0.0035
Septic shock, %	64	68	52	0.0035
Sequential Organ Failure Assessment score	9.6 ± 3.7	9.9 ± 3.8	8.9 ± 3.4	0.0158
Acute Physiology and Chronic Health Evaluation (APACHE II) score	22.1 ± 6.8	22.1 ± 7.0	22.1 ± 6.0	0.9888
Comorbidities, %				
Hypertension	56	48	78	<0.0001
History of myocardial infarction	6	2	19	<0.0001
Chronic obstructive pulmonary disease	18	18	20	0.5615
Renal dysfunction	11	9	18	0.0192
Noninsulin-dependent diabetes mellitus	9	7	15	0.0332
Insulin-dependent diabetes mellitus	11	9	19	0.0069
Chronic liver disease	7	7	7	1.0000
History of cancer	20	21	19	0.8891
History of stroke	5	4	11	0.0073
Recent surgical history, %				
Elective surgery	26	21	39	0.0005
Emergency surgery	54	56	49	0.2168
No history of surgery	20	23	12	0.0169
Site of infection, %				0.1339
Lung	60	57	70	
Abdomen	23	25	17	
Bone or soft tissue	5	5	2	
Surgical wound	2	2	1	
Urogenital	2	2	2	
Primary bacteremia	6	5	7	
Other	4	4	1	
Organ support, %				
Mechanical ventilation	90	91	86	0.1393
Use of vasopressor	64	68	52	0.0035
Renal replacement therapy	10	9	11	0.5697
Statin drugs, %				
Simvastatin	23		87	
Pravastatin	2		6	
Atorvastatin	1		6	
Fluvastatin	0		1	

The data are presented as the mean ± SD or as a percentage

Regarding comorbidities at baseline, the frequencies of several preexisting diseases were significantly higher in the patients on statin therapy (i.e., arterial hypertension, history of myocardial infarction, renal dysfunction, noninsulin-dependent diabetes mellitus, insulin-dependent diabetes mellitus, and history of stroke; Table 1). Furthermore, the number of patients with a recent surgical history also significantly differed between the two groups and there was no difference in the site of infection between the groups (Table 1). The patients on statin therapy required significantly less vasopressor therapy compared with those who were not on this therapy (52 % and 68 %, respectively; *P* = 0.0035; Table 1). We were able to follow all of these patients for a maximum of 90 days after sepsis onset.

### Outcomes

#### Mortality

**Fig. 2:**
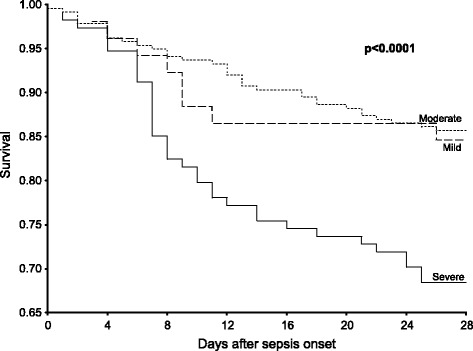
Kaplan-Meier survival analysis of 28-day survival according to acute respiratory distress syndrome (ARDS) severity. The Kaplan-Meier survival curves censored at day 28 for each ARDS group (mild, moderate, and severe). The mortality risk among the patients under study was higher for those with severe ARDS compared with those with mild and moderate ARDS (*P* <0.0001, log-rank test)

An analysis of the 28-day mortality risk of the patients according to ARDS severity revealed a significantly higher mortality rate among the patients with severe ARDS compared with those with mild or moderate ARDS (*P* <0.0001, log-rank test; Fig. 2).

**Fig. 3:**
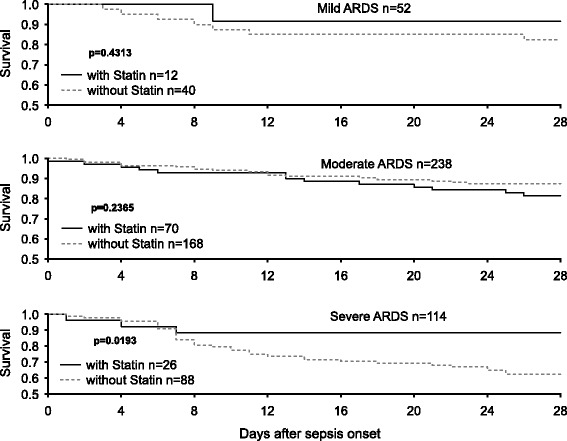
Kaplan-Meier survival analysis of 28-day survival according to statin therapy for the three acute respiratory distress syndrome (ARDS) groups. The Kaplan-Meier survival curves censored at day 28 for each ARDS group (mild, moderate, and severe) according to the presence of statin therapy. Treatment with statins only significantly impacted 28-day survival among the patients with severe sepsis-associated ARDS (*P* = 0.0193, log-rank test)

**Fig. 4:**
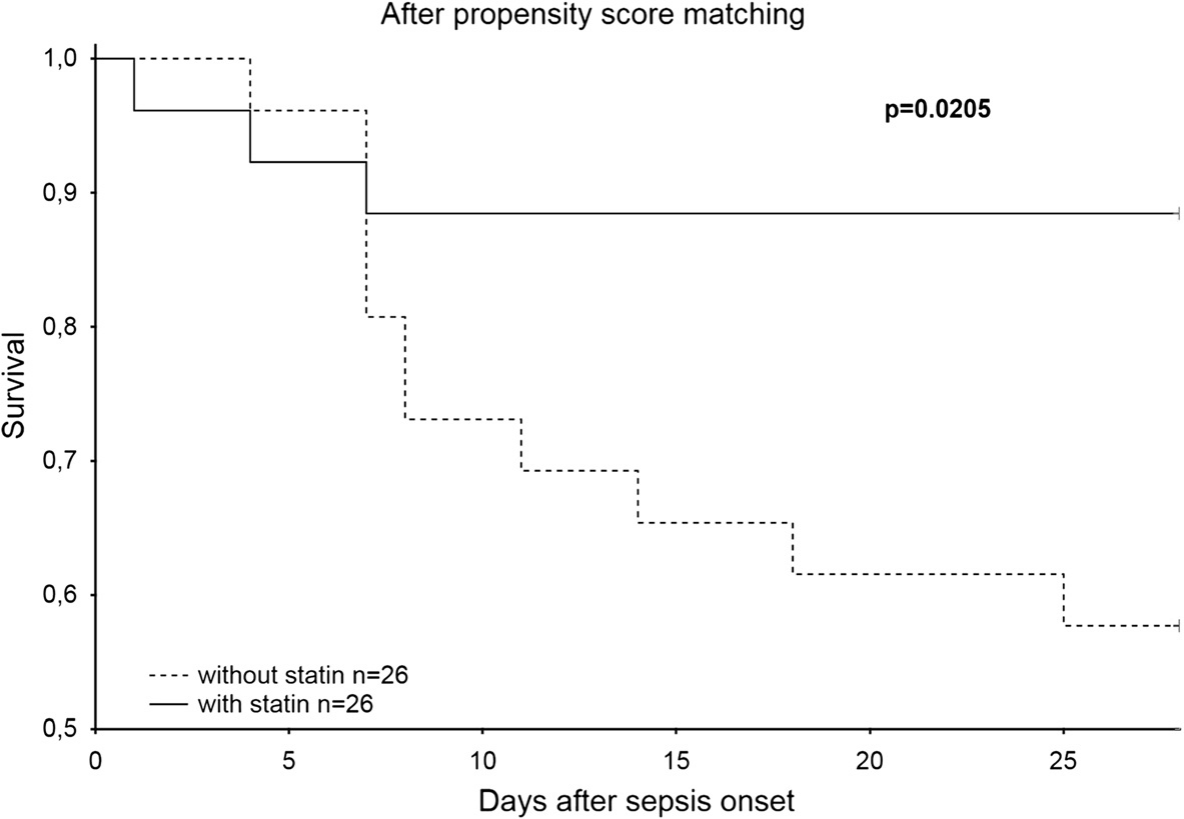
Kaplan-Meier survival analysis of 28-day survival after propensity score matching in the severe acute respiratory distress syndrome (ARDS) groups. The Kaplan-Meier survival curves censored at day 28 for severe ARDS group according to the presence of statin therapy. Treatment with statins significantly impacted 28-day survival among the patients with severe sepsis-associated ARDS (*P* = 0.0205, log-rank test)

Furthermore, to detect the impact of statin therapy on the outcome of sepsis-associated ARDS and its dependency on disease severity (mild, moderate, or severe), Kaplan-Meier survival analysis (mean outcome parameter) was performed to assess 28-day survival for these three groups of patients (Fig. 3). Treatment with statins significantly impacted the 28-day survival exclusively among the patients with severe sepsis-associated ARDS. Those with severe ARDS who were on statin therapy had a lower 28-day mortality rate compared with those who were not on this therapy (11.5 % and 37.5 %, respectively; *P* = 0.0193; Fig. 3). After performing propensity score matching, survival analysis remained significant with a marginal change (*P* = 0.0205 (after propensity score matching) vs. *P* = 0.0193 (without propensity score matching); Fig. 4). There were no differences in the 28-day mortality risk between the patients with statin therapy and those without therapy in the mild and moderate sepsis-associated ARDS groups (Fig. 3).

### Multivariate analysis

To exclude the effects of several confounders and baseline variables on 28-day survival among the patients with severe ARDS and to determine whether statin therapy is an independent prognostic factor for 28-day survival, the baseline patient characteristics in the severe ARDS group were analyzed according to the presence of statin therapy (Table 2). Subsequently, we performed multivariate Cox regression analysis and included statin therapy, potential confounders (age, gender, BMI, SOFA and APACHE II scores, and Corticosteroid therapy (given to patients for septic shock)) and covariates that varied at baseline (septic shock, arterial hypertension, and histories of stroke and elective surgery; Table 2). Multivariate Cox regression analysis revealed that the absence of statin therapy was an independent prognostic indicator of 28-day mortality risk (hazard ratio, 5.46; 95 % CI, 1.38–21.70; *P* = 0.0156) (Table 3).

**Table 2:** Severe acute respiratory distress syndrome (ARDS) patients’ baseline characteristics with regard to statin usage

	All	Severe ARDS		*P* value
	(n = 114)	without statins	with statins	
		(n = 88)	(n = 26)	
Age, years	61 ± 14	59 ± 15	67 ± 10	0.0159
Male, %	66	64	73	0.4822
Body mass index	30 ± 8	30 ± 9	30 ± 6	0.5590
Severity of sepsis				
Sepsis/severe sepsis, %	24	19	39	0.0644
Septic shock, %	76	81	62	0.0644
Sequential Organ Failure Assessment score	11.0 ± 3.5	11.2 ± 3.5	10.6 ± 3.4	0.2511
Acute Physiology and Chronic Health Evaluation (APACHE II) score	23.7 ± 6.7	23.4 ± 6.9	24.5 ± 5.7	0.3250
Comorbidities, %				
Hypertension	58	51	81	0.0073
History of myocardial infarction	4	2	12	0.0772
Chronic obstructive pulmonary disease	18	19	15	0.7787
Renal dysfunction	10	9	12	0.7114
Noninsulin-dependent diabetes mellitus	9	7	15	0.2323
Insulin-dependent diabetes mellitus	12	13	12	1.0000
Chronic liver disease	4	2	12	0.0772
History of cancer	19	22	12	0.3964
History of stroke	6	2	19	0.0066
Recent surgical history, %				
Elective surgery	25	19	42	0.0217
Emergency surgery	46	49	35	0.2635
No history of surgery	30	32	23	0.4704
Site of infection, %				0.5675
Lung	71	68	81	
Abdomen	11	13	4	
Bone or soft tissue	7	8	4	
Surgical wound	1	1	0	
Urogenital	1	1	0	
Primary bacteremia	7	6	12	
Other	3	3	0	
Organ support, %				
Mechanical ventilation	96	96	96	1.0000
Use of vasopressor	76	81	62	0.0644
Renal replacement therapy	11	10	12	1.0000
Statin drugs, %				
Simvastatin	20		96	
Pravastatin	1		4	

The data are presented as the mean ± SD or as a percentage

**Table 3:** Cox regression analysis of severe acute respiratory distress syndrome patients

Variable	Hazard ratio	95 % CI	*P* value
Age	1.00	0.98–1.04	0.5190
Male gender	1.13	0.55–2.34	0.7331
Body mass index	0.99	0.95–1.04	0.7431
Sequential Organ Failure Assessment	0.97	0.82–1.15	0.7618
Acute Physiology and Chronic Health Evaluation (APACHE II)	1.06	0.98–1.14	0.1287
Septic shock	0.74	0.24–2.33	0.6049
Arterial hypertension	1.38	0.66–2.91	0.3935
History of stroke	0.60	0.12–3.04	0.5358
Elective surgery	0.69	0.31–1.53	0.3556
Corticosteroid therapy	1.16	0.54–2.50	0.6983
No statin therapy	5.47	1.38–21.70	0.0156

### Disease severity

Analysis of SOFA scores obtained during the ICU stays of the patients with severe ARDS revealed significantly higher scores among those without statin therapy for three organ-specific SOFA scores (the cardiovascular, central nervous system, and hepatic scores; Table 4). Furthermore, the patients with severe ARDS who received statin therapy had significantly more vasopressor-free days compared with those without this therapy (13 ± 7 and 9 ± 7, respectively; *P* = 0.0034). The severe ARDS patients on statin therapy required less ECMO therapy and had more ECMO-free days compared with those without statin therapy (18 ± 9 and 15 ± 9, respectively; *P* = 0.0873; Table 4). Distribution of infection types is shown in Table 5. Additional results regarding disease severity, microbiological findings and anti-infective agents were added to Additional file 1: Tables S1–S4.

**Table 4:** Disease severity among patients with severe acute respiratory distress syndrome (ARDS) according to statin therapy

	All	Severe ARDS		*P* value
	(n = 114)	without statins	with statins	
		(n = 88)	(n = 26)	
Sequential Organ Failure Assessment (SOFA)	9.0 ± 3.7	9.3 ± 3.9	7.7 ± 2.6	0.1051
SOFA-Respiratory score	2.6 ± 0.6	2.6 ± 0.6	2.6 ± 0.6	0.9677
SOFA-Cardiovascular score	2.0 ± 1.1	2.1 ± 1.1	1.5 ± 1.0	0.0063
SOFA-Central nervous System score	2.4 ± 1.0	2.5 ± 1.0	2.0 ± 1.0	0.0203
SOFA-Renal score	1.0 ± 1.2	0.9 ± 1.2	1.1 ± 1.0	0.2110
SOFA-Coagulation score	0.5 ± 0.7	0.6 ± 0.8	0.3 ± 0.4	0.2734
SOFA-Hepatic score	0.5 ± 0.7	0.5 ± 0.8	0.3 ± 0.5	0.0218
Mortality analysis, %				
Death at day 28	32	38	12	0.0153
Death at day 90	42	47	27	0.1126
Length of stay in ICU, days	20 ± 15	19 ± 15	22 ± 16	0.4290
Organ support-free days				
Vasopressor-free days	10 ± 7	9 ± 7	13 ± 7	0.0034
Ventilator-free days	3 ± 3	2 ± 3	3 ± 3	0.1824
Dialysis-free days	15 ± 8	14 ± 8	17 ± 8	0.2532
Extracorporeal membrane oxygenation-free days	15 ± 9	15 ± 9	18 ± 9	0.0873
Inflammatory values				
Leucocytes (1000/μL)	13 ± 5	13 ± 5	12 ± 4	0.5168
C-reactive protein (mg/L) (n)	155 ± 84 (46)	166 ± 85 (33)	125 ± 79 (13)	0.1400
Procalcitonin (ng/dL) (n)	6.4 ± 14.0 (109)	7.3 ± 15.1 (85)	3.2 ± 9.5 (24)	0.0743
Kidney values				
Urine output (mL/day)	2977 ± 1442	2911 ± 1440	3201 ± 1457	0.5659
Urine output (mL/kg/h)	1.5 ± 0.8	1.4 ± 0.7	1.5 ± 0.8	0.7356
Creatinine (mg/dL)	1.4 ± 0.9	1.3 ± 1.0	1.6 ± 0.8	0.0363
Liver values				
AST (IU/L) (n)	391 ± 1248 (81)	472 ± 1395 (64)	85 ± 80 (17)	0.0611
ALT (IU/L) (n)	135 ± 288 (113)	157 ± 324 (87)	63 ± 57 (26)	0.1257
Bilirubin (mg/dL)	1.5 ± 2.7	1.7 ± 3.0	0.8 ± 0.7	0.0511

The data are presented as the mean ± SD or as a percentage

**Table 5:** Infection types during the observational period

Infection type	Severe acute respiratory distress syndrome		*P* value
	without statins	with statins	
	(n = 88)	(n = 26)	
Gram-negative	71 %	65 %	0.6347
Gram-positive	78 %	96 %	0.0407
Fungal	61 %	81 %	0.0985
Viral	10 %	15 %	0.4892

### Inflammatory parameters

The patients with statin therapy in the severe ARDS group had reduced C-reactive protein (CRP) levels compared with those without this therapy (125 ± 79 and 166 ± 85, respectively; *P* = 0.1400). Furthermore, the severe ARDS patients with statin therapy had lower levels of procalcitonin compared with those without therapy (3.2 ± 9.5 and 7.3 ± 15.1, respectively; *P* = 0.0743; Table 4).

## Discussion

This prospective observational study addresses the question of whether statin therapy in patients with sepsis-associated ARDS is associated with 28-day survival, according to the new Berlin definition of ARDS severity (mild, moderate, and severe). The main finding of this investigation was that patients with severe sepsis-associated ARDS who received statin therapy had a significantly better 28-day survival rate compared with those without this therapy.

The observed beneficial effect of statin therapy on 28-day survival, which was nearly exclusively observed in the patients with severe sepsis-associated ARDS, is in accordance with recent studies showing that the various ARDS subgroups (mild, moderate, and severe) are associated with distinct histopathological features that may impact the therapeutic potentials of or responses to ARDS-specific treatments [28]. According to Thille et al. [28], patients with severe ARDS comprise a homogeneous group characterized by a high proportion of diffuse alveolar damage compared with those with mild or moderate ARDS. Diffuse alveolar damage is accompanied by a severe inflammatory state; therefore, patients with severe ARDS are more likely to benefit from the anti-inflammatory, pleiotropic effects of statin therapy. Our results confirm previous reports of the beneficial effects of statin therapy continuation on the survival of patients with sepsis [29, 30]. In a recent multicenter prospective study that included patients with severe sepsis, Kruger et al. [29] showed that the continuation of atorvastatin in patients with severe sepsis who have received statin pretreatment is associated with improved survival.

Our findings of better survival among the patients with severe ARDS who received continuous statin therapy are of particular importance because there is currently no specific effective treatment that improves the clinical course of patients with severe ARDS. Current treatment modalities focus on symptomatic treatments, including mechanical ventilation and organ support. This evidence of a beneficial impact of statin continuation on severe ARDS patients suggests that statin therapy should be continued for all critically ill patients who are at high risk of developing sepsis or infection, i.e., those undergoing surgical procedures with a high infection risk. Furthermore, according to our investigation, preventive statin treatment in patients with a high predisposition to sepsis or sepsis-associated ARDS may be of potential therapeutic significance and should be addressed in future studies. Analogous to evidence demonstrated by previous cardiovascular studies that a statin treatment duration of 8 to 12 weeks is needed to achieve pleotropic effects [31–33], we believe that patients with sepsis-associated ARDS must be pretreated with statins for 8 to 12 weeks to achieve beneficial effects in this patient group.

Moreover, we found that the beneficial impact of statin therapy was accompanied by significantly lower organ-specific SOFA scores in three organ systems (the cardiovascular, central nervous, and hepatic systems) among the patients on statin therapy (Table 4). The observed lower cardiovascular scores might have been due to anti-inflammatory effects of the statin therapy, which inhibited the release of inflammatory mediators and resulted in less vasodilatation in the patients in this group [5]. In accordance with the lower cardiovascular SOFA scores, severe sepsis-associated ARDS patients also had significantly more vasopressor-free days compared with those who were not on statin therapy (Table 4). Similarly, the observed lower hepatic SOFA scores can be explained by the fact that statin therapy has a beneficial effect on liver function [5]. As shown by Arnaud et al. [34], statins exert direct anti-inflammatory effects on hepatocytes by reducing IL-6-induced CRP production.

Although they did not reach statistical significance, the lower inflammatory, CRP, and procalcitonin levels among the patients on continuous statin therapy (Table 4) are in accordance with previous observations showing an effect of statin therapy on reducing inflammatory parameters in patients with severe sepsis [8, 29] and in a human model of acute lung injury [6].

There are some limitations to this study. This is an observational study; therefore, it did not apply the ideal methodology for assessing the effects of a drug. A future randomized controlled (RCT) study would be much more suitable, although this study does provide impetus for potential studies to examine the use of statins in severe ARDS. This type of RCT would be very difficult to conduct because the duration of statin pretreatment and the dose required to achieve beneficial effects on the clinical course for patients with severe sepsis-associated ARDS are unknown. A further potential limitation is that plasma statin levels were not measured. Therefore, we cannot rule out the fact that statin levels were not within the therapeutic range. However, according to previous investigations, high plasma levels of statins are achieved (especially in critically ill patients in the ICU) even after the administration of a single dose [8, 35]. Moreover, the plasma level of statins required to induce anti-inflammatory effects is not known. Furthermore, because no previous investigations have addressed the effects of statin therapy on ARDS according to disease severity, we were unable to conduct power calculations at the beginning of the study to estimate a sample size with sufficient power. However, ad hoc power analysis yielded a power of 0.87, according to our observation of 11.5 % mortality in the severe ARDS patients on statin therapy compared with 37.5 % mortality in those who were not on this therapy. Therefore, our investigated cohort of 404 patients with sepsis-associated ARDS was sufficient to address our hypotheses.

To the best of our knowledge, this investigation is the first to evaluate the impact of statin therapy on the survival of patients with sepsis-associated ARDS according to the new Berlin definition of ARDS. Our results provide evidence that confirms the valuable and important application of the new Berlin definition in guiding the therapy of critically ill ARDS patients and underscores the potential therapeutic benefits of statins in this high-risk patient cohort. Further study is warranted to elucidate the potential beneficial effects of statin therapy in patients with severe ARDS.

## Conclusions

This prospective observational cohort study has confirmed the hypothesis that statin therapy does improve the clinical course of sepsis-associated ARDS depending on disease severity (mild, moderate, or severe). Therefore, the use of continuous statin therapy in sepsis patients with a prior history of receiving this therapy who develop severe ARDS has an independent beneficial impact on 28-day survival. Further study is warranted to explain this potential effect.

## Additional file

Additional file 1:
**The additional file 1 includes information about vital parameters, laboratory parameters, kidney parameters and inflammation values, recorded microbiological findings and anti-infective agents.**

## Abbreviations

APACHE: Acute physiology and chronic health evaluation
ARDS: Acute respiratory distress syndrome
BMI: Body mass index
CRP: C-reactive protein
ECMO: Extracorporeal membrane oxygenation
ICU: Intensive care unit
SOFA: Sequential organ failure assessment
