# Supplementary material for: Impact of statin therapy on mortality in patients with sepsis-associated acute respiratory distress syndrome (ARDS) depends on ARDS severity: a prospective observational cohort study
Source: BMC Med. 2015 Jun 1;13:128. doi: 10.1186/s12916-015-0368-6 (PMC4462111; doi:10.1186/s12916-015-0368-6)
Supplement: Additional file 1: — The additional file 1 includes information about vital parameters, laboratory parameters, kidney parameters and inflammation values, recorded microbiological findings and anti-infective agents. [file 12916_2015_368_MOESM1_ESM.docx]

**Additional file 1; Table 1: Vital parameters, laboratory parameters, kidney parameters and inflammation values**

|  | All  n=114 | without Statin  n=88 | with Statin  n=26 | P value |
| --- | --- | --- | --- | --- |
| vital parameters, mean ± SD |  |  |  |  |
| Temperature (°C), max | 38.0±0.5 | 38.1±0.5 | 37.9±0.6 | 0.2496 |
| Temperature (°C), min | 36.9±0.5 | 36.9±0.5 | 36.9±0.6 | 0.6244 |
| Heart rate (bpm), max | 105±14 | 105±15 | 105±11 | 0.7561 |
| Heart rate (bpm), min | 75±12 | 76±12 | 74±11 | 0.6730 |
| MAP (mmHg), max | 99±11 | 99±12 | 100±10 | 0.6031 |
| MAP (mmHg), min | 65±9 | 65±9 | 64±8 | 0.7204 |
| Vasopressor (µg/kg/min) (n) | 13±12  (103) | 14±12  (83) | 11±13  (20) | 0.2157 |
| laboratory parameters, mean ± SD |  |  |  |  |
| Lactate (mmol/l) | 2.1±1.5 | 2.2±1.6 | 1.7±0.8 | 0.3393 |
| Thrombocytes (1000/µl) | 270±149 | 262±145 | 299±156 | 0.2537 |
| Quick (%) (n) | 81±15  (114) | 81±16  (88) | 82±14  (26) | 0.8236 |

MAP=Mean arterial pressure; The data are presented as the mean±SD or percentages. Min and Max indicate the lowest/highest value that has been recorded daily within the observation period.

**Additional file 1; Table 2. Recorded microbiological findings**

|  | All  n=114 | without Statin  n=88 | with Statin  n=26 |
| --- | --- | --- | --- |
| Bacteria |  |  |  |
| Gram-negative, n (%) |  |  |  |
| Acinetobacter genomospecies 3 | 1 (0.9) | 1 (1.1) | 0 (0.0) |
| Acinetobacter iwoffii | 1 (0.9) | 1 (1.1) | 0 (0.0) |
| Bacteroides fragilis | 3 (2.6) | 2 (2.3) | 1 (3.9) |
| Bacteroides species | 1 (0.9) | 1 (1.1) | 0 (0.0) |
| Bacteroides thetaiotaomicron | 4 (3.5) | 3 (3.4) | 1 (3.9) |
| Bacteroides uniformis | 1 (0.9) | 1 (1.1) | 0 (0.0) |
| Chlamydia pneumoniae IgA | 5 (4.4) | 4 (4.6) | 1 (3.9) |
| Chlamydia pneumoniae lgG | 2 (1.8) | 1 (1.1) | 1 (3.9) |
| Chlamydophila pneumoniae | 4 (3.5) | 3 (3.4) | 1 (3.9) |
| Citrobacter freundii | 2 (1.8) | 1 (1.1) | 1 (3.9) |
| Citrobacter koseri | 5 (4.4) | 5 (5.7) | 0 (1.0) |
| Enterobacter aerogenes | 2 (1.8) | 2 (2.3) | 0 (0.0) |
| Enterobacter cloacae | 8 (7.0) | 5 (5.7) | 3 (11.5) |
| Enterobacteriaceae | 1 (0.9) | 1 (1.1) | 0 (0.0) |
| Escherichia coli | 34 (29.8) | 28 (31.8) | 6 (23.1) |
| Haemophilus influenza | 7 (6.1) | 7 (8.0) | 0 (0.0) |
| Haemophilus parainfluenzae | 3 (2.6) | 3 (3.4) | 0 (0.0) |
| Hafnia alvei | 1 (0.9) | 1 (1.1) | 0 (0.0) |
| Klebsiella oxytoca | 4 (3.5) | 3 (3.4) | 1 (3.9) |
| Klebsiella pneumoniae | 8 (7.0) | 4 (4.6) | 4 (15.4) |
| Morgonella morganii | 2 (1.8) | 2 (2.3) | 0 (0.0) |
| Pantoea agglomerans | 3 (2.6) | 3 (3.4) | 0 (0.0) |
| Proteus mirabilis | 5 (4.4) | 3 (3.4) | 2 (7.7) |
| Pseudomonas aeruginosa | 14 (12.3) | 10 (11.4) | 4 (15.4) |
| Raoultella ornithinolytica | 1 (0.9) | 1 (1.0) | 0 (0.0) |
| Serratia marcescens | 12 (10.5) | 8 (9.1) | 4 (15.4) |
| Serratia fonticola | 1 (0.9) | 1 (1.1) | 0 (0.0) |
| Serratia liquefaciens | 1 (0.9) | 1 (1.1) | 0 (0.0) |
| Stenotrophomonas maltophila | 1 (0.9) | 1 (1.1) | 0 (0.0) |
| Gram-positive, n (%) |  |  |  |
| Actinomyces species | 1 (0.9) | 0 (0.0) | 1 (3.9) |
| Bacillus mycoides | 1 (0.9) | 1 (1.1) | 0 (0.0) |
| Bacillus cereus | 2 (1.8) | 2 (2.3) | 0 (0.0) |
| Bacillus species | 1 (0.9) | 1 (1.1) | 0 (0.0) |
| Clostridium difficile | 3 (2.6) | 2 (2.3) | 1 (3.9) |
| Clostridium innocuum | 1 (0.9) | 1 (1.1) | 0 (0.0) |
| Corynebacterium amycolatum | 1 (0.9) | 0 (0.0) | 1 (3.9) |
| Corynebacterium species | 3 (2.6) | 2 (2.3) | 1 (3.9) |
| Enterococcus avium | 1 (0.9) | 1 (1.1) | 0 (0.0) |
| Enterococcus faecalis | 18 (15.8) | 15 (17.1) | 3 (11.5) |
| Enterococcus faecium | 18 (15.8) | 13 (14.8) | 5 (19.2) |
| Enterococcus species | 29 (25.4) | 19 (21.6) | 10 (38.5) |
| Coagulase negative Staphylococci | 10 (8.8) | 8 (9.1) | 2 (7.7) |
| Gemella morbillorum | 1 (0.9) | 1 (1.1) | 0 (0.0) |
| Gram positive Coccus | 21 (18.4) | 16 (18.1) | 5 (19.2) |
| Lactobacillus paracasei | 2 (1.8) | 2 (2.3) | 0 (0.0) |
| MRSA | 5 (4.4) | 2 (2.3) | 3 (11.5) |
| Propionibacterium acnes | 1 (0.9) | 1 (1.1) | 0 (0.0) |
| Rothia mucilaginosa | 2 (1.9) | 1 (1.1) | 1 (3.9) |
| Staphyloccocus aureus | 22 (19.3) | 14 (15.9) | 8 (30.8) |
| Staphylococcus capitis | 6 (5.3) | 5 (5.7) | 1 (3.9) |
| Staphylococcus epidermidis | 41 (36.0) | 28 (31.8) | 13 (50.0) |
| Staphylococcus haemolyticus | 4 (3.5) | 1 (1.1) | 3 (11.5) |
| Staphylococcus hominis | 1 (0.9) | 1 (1.1) | 0 (0.0) |
| Staphylococcus wameri | 1 (0.9) | 1 (1.1) | 0 (0.0) |
| Streptococcus agalactiae | 2 (1.8) | 2 (2.3) | 0 (0.0) |
| Streptococcus anginosus | 2 (1.8) | 2 (2.3) | 0 (0.0) |
| Streptococcus pneumonia | 2 (1.8) | 2 (2.3) | 0 (0.0) |
| Streptococcus viridans | 4 (3.5) | 3 (3.4) | 1 (3.9) |
| Streptococcus parasanguinus | 1 (0.9) | 1 (1.1) | 0 (0.0) |
| Fungi, n (%) |  |  |  |
| Aspergillus fumigatus | 2 (1.8) | 1 (1.1) | 1 (3.9) |
| Candida albicans | 42 (36.8) | 28 (31.8) | 14 (53.9) |
| Candida dubliniensis | 3 (2.6) | 3 (3.4) | 0 (0.0) |
| Candida glabrata | 19 (16.7) | 13 (14.8) | 6 (23.1) |
| Candida guilliermondii | 1 (0.9) | 1 (1.1) | 0 (0.0) |
| Candida IgG | 1 (0.9) | 1 (1.1) | 0 (0.0) |
| Candida IgA | 2 (1.8) | 1 (1.1) | 1 (3.9) |
| Candida Antigen | 4 (3.5) | 3 (3.4) | 1 (3.9) |
| Candida krusei | 6 (5.3) | 4 (4.6) | 2 (7.7) |
| Candida lusitaniae | 2 (1.8) | 1 (1.1) | 1 (3.9) |
| Candida parapsilosis | 4 (3.5) | 3 (3.4) | 1 (3.9) |
| Candida tropicalis | 9 (7.9) | 9 (10.2) | 0 (0.0) |
| Viruses, n (%) |  |  |  |
| Adenovirus-Ag-IFT | 1 (0.9) | 1 (1.1) | 0 (0.0) |
| Anti-HBS IgG | 2 (1.8) | 1 (1.1) | 1 (3.9) |
| CMV | 3 (2.6) | 2 (2.3) | 1 (3.9) |
| H1N1 (2009 RNA) | 1 (0.9) | 1 (1.1) | 0 (0.0) |
| H1N1 DNA | 4 (3.5) | 3 (3.4) | 1 (3.9) |
| HSV | 1 (0.9) | 1 (1.1) | 0 (0.0) |
| RS-Virusantigen IFT | 1 (0.9) | 1 (1.1) | 0 (0.0) |
| EBV | 2 (1.8) | 1 (1.1) | 1 (3.9) |
| Others, n (%) |  |  |  |
| Mycoplasma Pneumonia | 1 (0.9) | 1 (1.1) | 0 (0.0) |

CMV: Cytomegalovirus; MRSA: Methicillin-resistant Staphylococcus aureus; RS-Virus: Respiratory Syncytial Virus.

**Additional file 1; Table 3. Anti-infective agents**

|  | All  n=114 | without Statin  n=88 | with Statin  n=26 |
| --- | --- | --- | --- |
| Antibiotics, n (%) |  |  |  |
| Penicillins | 74 (65) | 58 (66) | 16 (62) |
| Aminopenicillins | 14 (12) | 10 (11) | 4 (15) |
| 1. generation cephalosporines | 16 (14) | 9 (10) | 7 (27) |
| 2. generation cephalosporines | 5 (4) | 2 (2) | 3 (12) |
| 3. generation cephalosporines | 18 (16) | 13 (15) | 5 (19) |
| Carbapenems | 91 (80) | 72 (82) | 19 (73) |
| Macrolides | 52 (46) | 42 (48) | 10 (39) |
| Aminoglycosides | 10 (9) | 8 (9) | 2 (8) |
| Fluorchinolones | 21 (18) | 14 (16) | 7 (27) |
| Imidazoles | 4 (4) | 3 (3) | 1 (4) |
| Glycopeptides | 41 (36) | 29 (33) | 12 (46) |
| Lipopeptides | 2 (2) | 1 (1) | 1 (4) |
| Lincosamides | 8 (7) | 7 (8) | 1 (4) |
| Oxazolidinones | 51 (45) | 39 (44) | 12 (46) |
| Glycylcyclines | 1 (1) | 1 (1) | 0 (0) |
| Rifampicin | 1 (1) | 1 (1) | 0 (0) |
| Sulfamethoxazol/Trimethoprim | 4 (4) | 3 (3) | 1 (4) |
| Antifungals, n (%) |  |  |  |
| Echinocandin | 27 (24) | 21 (24) | 6 (23) |
| Triazole derivatives | 24 (21) | 18 (21) | 6 (23) |
| Polyene | 3 (3) | 2 (2) | 1 (4) |
| Antivirals, n (%) |  |  |  |
| Aciclovir | 1 (1) | 1 (1) | 0 (0) |
| Oseltamivir | 4 (4) | 3 (3) | 1 (4) |

**Additional file 1; Table 4. Distribution of hospital-acquired infections**

| pathogens | Statin (n=108) | No Statin (n=296) | in total (n) | Statin (n=108) (%) | No Statin (n=296) (%) | in total (%) |
| --- | --- | --- | --- | --- | --- | --- |
| Acinetobacter calcoacticus | 1 | 2 | 3 | 0.93 | 0.68 | 0.74 |
| Acinetobacter genomospecies 3 | 1 | 6 | 7 | 0.93 | 2.03 | 1.73 |
| Acinetobacter lwoffii |  | 1 | 1 | 0.00 | 0.34 | 0.25 |
| Actinomyces species | 1 | 1 | 2 | 0.93 | 0.34 | 0.50 |
| Adenovirus-Ag-IFT | 1 | 1 | 2 | 0.93 | 0.34 | 0.50 |
| Aerococcus urinae | 1 |  | 1 | 0.93 | 0.00 | 0.25 |
| Aggregatibacter segnis |  | 1 | 1 | 0.00 | 0.34 | 0.25 |
| Alistipes finegoldii |  | 1 | 1 | 0.00 | 0.34 | 0.25 |
| Anti-HBS (IgG) | 1 | 2 | 3 | 0.93 | 0.68 | 0.74 |
| Aspergillus flavus |  | 1 | 1 | 0.00 | 0.34 | 0.25 |
| Aspergillus fumigatus | 2 | 4 | 6 | 1.85 | 1.35 | 1.49 |
| Aspergillus spezies | 1 | 2 | 3 | 0.93 | 0.68 | 0.74 |
| Bacillus cereus |  | 8 | 8 | 0.00 | 2.70 | 1.98 |
| Bacillus circulans |  | 1 | 1 | 0.00 | 0.34 | 0.25 |
| Bacillus mycoides |  | 4 | 4 | 0.00 | 1.35 | 0.99 |
| Bacillus species |  | 2 | 2 | 0.00 | 0.68 | 0.50 |
| Bacillus thuringiensis |  | 2 | 2 | 0.00 | 0.68 | 0.50 |
| Bacteroides fragilis | 5 | 7 | 12 | 4.63 | 2.36 | 2.97 |
| Bacteroides ovaters |  | 2 | 2 | 0.00 | 0.68 | 0.50 |
| Bacteroides species | 1 | 3 | 4 | 0.93 | 1.01 | 0.99 |
| Bacteroides thetaiotaomicron | 2 | 6 | 8 | 1.85 | 2.03 | 1.98 |
| Bacteroides uniformis |  | 4 | 4 | 0.00 | 1.35 | 0.99 |
| Bordetella bronchiseptika |  | 1 | 1 | 0.00 | 0.34 | 0.25 |
| Burkholderia gladioli |  | 1 | 1 | 0.00 | 0.34 | 0.25 |
| Candida antigen | 5 | 20 | 25 | 4.63 | 6.76 | 6.19 |
| Candida albicans | 32 | 108 | 140 | 29.63 | 36.49 | 34.65 |
| Candida dubliniensis |  | 4 | 4 | 0.00 | 1.35 | 0.99 |
| Candida glabrata | 14 | 39 | 53 | 12.96 | 13.18 | 13.12 |
| Candida guilliermondii |  | 2 | 2 | 0.00 | 0.68 | 0.50 |
| Candida IgA | 7 | 8 | 15 | 6.48 | 2.70 | 3.71 |
| Candida IgG | 6 | 10 | 16 | 5.56 | 3.38 | 3.96 |
| Candida krusei | 3 | 10 | 13 | 2.78 | 3.38 | 3.22 |
| Candida lusitaniae | 1 | 1 | 2 | 0.93 | 0.34 | 0.50 |
| Candida palmioleophila | 1 | 1 | 2 | 0.93 | 0.34 | 0.50 |
| Candida parapsilosis | 2 | 7 | 9 | 1.85 | 2.36 | 2.23 |
| Candida tropicalis | 8 | 25 | 33 | 7.41 | 8.45 | 8.17 |
| Chlamydia pneumoniae IgA | 3 | 8 | 11 | 2.78 | 2.70 | 2.72 |
| Chlamydia pneumoniae lgG | 3 | 2 | 5 | 2.78 | 0.68 | 1.24 |
| Chlamydophila pneumoniae | 1 | 7 | 8 | 0.93 | 2.36 | 1.98 |
| Citrobacter braakii | 2 |  | 2 | 1.85 | 0.00 | 0.50 |
| Citrobacter freundii | 3 | 6 | 9 | 2.78 | 2.03 | 2.23 |
| Citrobacter koseri | 2 | 5 | 7 | 1.85 | 1.69 | 1.73 |
| Clostridium difficile | 2 | 8 | 10 | 1.85 | 2.70 | 2.48 |
| Clostridium innocuum |  | 2 | 2 | 0.00 | 0.68 | 0.50 |
| Clostridium perfringens |  | 2 | 2 | 0.00 | 0.68 | 0.50 |
| Clostridium tertium |  | 1 | 1 | 0.00 | 0.34 | 0.25 |
| CMV | 5 | 2 | 7 | 4.63 | 0.68 | 1.73 |
| Coliform rods |  | 1 | 1 | 0.00 | 0.34 | 0.25 |
| Corynebacterium species | 3 | 4 | 7 | 2.78 | 1.35 | 1.73 |
| Corynebacterium amycolatum | 2 | 1 | 3 | 1.85 | 0.34 | 0.74 |
| EBV-EBNA1 | 1 |  | 1 | 0.93 | 0.00 | 0.25 |
| EBV-VCA | 2 | 2 | 4 | 1.85 | 0.68 | 0.99 |
| Enterobacter aerogenes |  | 3 | 3 | 0.00 | 1.01 | 0.74 |
| Enterobacter asburiae | 1 | 1 | 2 | 0.93 | 0.34 | 0.50 |
| Enterobacter cloacae | 12 | 27 | 39 | 11.11 | 9.12 | 9.65 |
| Enterobacteriaceae |  | 1 | 1 | 0.00 | 0.34 | 0.25 |
| Enterococcus avium |  | 3 | 3 | 0.00 | 1.01 | 0.74 |
| Enterococcus faecalis | 14 | 57 | 71 | 12.96 | 19.26 | 17.57 |
| Enterococcus faecium | 23 | 59 | 82 | 21.30 | 19.93 | 20.30 |
| Enterococcus gallinarum | 1 | 1 | 2 | 0.93 | 0.34 | 0.50 |
| Enterococcus mundtii |  | 1 | 1 | 0.00 | 0.34 | 0.25 |
| Enterococcus species | 25 | 66 | 91 | 23.15 | 22.30 | 22.52 |
| ESBL E. coli | 4 | 3 | 7 | 3.70 | 1.01 | 1.73 |
| Escherichia coli | 29 | 88 | 117 | 26.85 | 29.73 | 28.96 |
| Fusobacterium necrophorum |  | 1 | 1 | 0.00 | 0.34 | 0.25 |
| Gemella morbillorum | 1 | 1 | 2 | 0.93 | 0.34 | 0.50 |
| Gram-positive cocci | 16 | 44 | 60 | 14.81 | 14.86 | 14.85 |
| H1N1 RNA |  | 2 | 2 | 0.00 | 0.68 | 0.50 |
| H1N1 DNA | 1 | 3 | 4 | 0.93 | 1.01 | 0.99 |
| Haemophilus influenza | 2 | 21 | 23 | 1.85 | 7.09 | 5.69 |
| Haemophilus parainfluenzae |  | 6 | 6 | 0.00 | 2.03 | 1.49 |
| Hafnia alvei |  | 5 | 5 | 0.00 | 1.69 | 1.24 |
| HSV | 3 | 4 | 7 | 2.78 | 1.35 | 1.73 |
| Klebsiella ornitholytica | 1 | 1 | 2 | 0.93 | 0.34 | 0.50 |
| Klebsiella oxytoca | 6 | 9 | 15 | 5.56 | 3.04 | 3.71 |
| Klebsiella pneumoniae | 13 | 29 | 42 | 12.04 | 9.80 | 10.40 |
| Coagulase-negative staphylococci | 11 | 28 | 39 | 10.19 | 9.46 | 9.65 |
| Lactobacillus paracasei |  | 3 | 3 | 0.00 | 1.01 | 0.74 |
| Lactobacillus rhamnosus |  | 6 | 6 | 0.00 | 2.03 | 1.49 |
| Legionella species |  | 2 | 2 | 0.00 | 0.68 | 0.50 |
| Morganella morganii | 1 | 10 | 11 | 0.93 | 3.38 | 2.72 |
| MRSA | 6 | 9 | 15 | 5.56 | 3.04 | 3.71 |
| Mycoplasma pneumoniae |  | 2 | 2 | 0.00 | 0.68 | 0.50 |
| Pantoea agglomerans |  | 4 | 4 | 0.00 | 1.35 | 0.99 |
| Peptostreptococcus species |  | 2 | 2 | 0.00 | 0.68 | 0.50 |
| Propionibacterium acnes |  | 4 | 4 | 0.00 | 1.35 | 0.99 |
| Propionibacterium species |  | 1 | 1 | 0.00 | 0.34 | 0.25 |
| Proteus mirabilis | 12 | 27 | 39 | 11.11 | 9.12 | 9.65 |
| Proteus penneri |  | 1 | 1 | 0.00 | 0.34 | 0.25 |
| Proteus species | 3 |  | 3 | 2.78 | 0.00 | 0.74 |
| Proteus vulgaris | 2 | 2 | 4 | 1.85 | 0.68 | 0.99 |
| Pseudomonas aeruginosa | 17 | 45 | 62 | 15.74 | 15.20 | 15.35 |
| Pseudomonas geniculata |  | 1 | 1 | 0.00 | 0.34 | 0.25 |
| Pseudomonas korrensis |  | 1 | 1 | 0.00 | 0.34 | 0.25 |
| Pseudomonas putida |  | 2 | 2 | 0.00 | 0.68 | 0.50 |
| Raoultella ornithinolytica | 2 | 3 | 5 | 1.85 | 1.01 | 1.24 |
| Raoultella planticola | 1 |  | 1 | 0.93 | 0.00 | 0.25 |
| Rotavirus |  | 1 | 1 | 0.00 | 0.34 | 0.25 |
| Rothia mucilaginosa | 1 | 1 | 2 | 0.93 | 0.34 | 0.50 |
| Ruminococcus gnavus |  | 1 | 1 | 0.00 | 0.34 | 0.25 |
| Serratia fonticola |  | 1 | 1 | 0.00 | 0.34 | 0.25 |
| Serratia liquefaciens |  | 2 | 2 | 0.00 | 0.68 | 0.50 |
| Serratia marcescens | 10 | 18 | 28 | 9.26 | 6.08 | 6.93 |
| Staphyloccocus aureus | 24 | 71 | 95 | 22.22 | 23.99 | 23.51 |
| Staphyloccocus pyogenes |  | 1 | 1 | 0.00 | 0.34 | 0.25 |
| Staphylococcus capitis | 3 | 13 | 16 | 2.78 | 4.39 | 3.96 |
| Staphylococcus epidermidis | 46 | 100 | 146 | 42.59 | 33.78 | 36.14 |
| Staphylococcus haemolyticus | 6 | 16 | 22 | 5.56 | 5.41 | 5.45 |
| Staphylococcus hominis | 2 | 8 | 10 | 1.85 | 2.70 | 2.48 |
| Staphylococcus lugdunensis |  | 1 | 1 | 0.00 | 0.34 | 0.25 |
| Staphylococcus wameri | 1 | 2 | 3 | 0.93 | 0.68 | 0.74 |
| Stenotrophomonas maltophila | 5 | 10 | 15 | 4.63 | 3.38 | 3.71 |
| Streptococcus agalactiae | 1 | 9 | 10 | 0.93 | 3.04 | 2.48 |
| Streptococcus anginosus | 4 | 8 | 12 | 3.70 | 2.70 | 2.97 |
| Streptococcus constellatus | 3 | 11 | 14 | 2.78 | 3.72 | 3.47 |
| Streptococcus cristatus |  | 1 | 1 | 0.00 | 0.34 | 0.25 |
| Streptococcus parasanguis |  | 2 | 2 | 0.00 | 0.68 | 0.50 |
| Streptococcus pneumonia | 2 | 6 | 8 | 1.85 | 2.03 | 1.98 |
| Streptococcus salivarius |  | 1 | 1 | 0.00 | 0.34 | 0.25 |
| Streptococcus viridans | 4 | 10 | 14 | 3.70 | 3.38 | 3.47 |
| Toxoplasmosis IgG |  | 1 | 1 | 0.00 | 0.34 | 0.25 |
| VZV |  | 1 | 1 | 0.00 | 0.34 | 0.25 |
| In total | 444 | 1234 | 1678 | 411.11 | 416.89 | 415.35 |
